# Long-term bat abundance in sagebrush steppe

**DOI:** 10.1038/s41598-018-30402-z

**Published:** 2018-08-16

**Authors:** Jericho C. Whiting, Bill Doering, Gary Wright, Devin K. Englestead, Justin A. Frye, Todd Stefanic, Brent J. Sewall

**Affiliations:** 1 Department of Biology, Brigham Young University-Idaho, 116 Benson Building, Rexburg, Idaho USA; 2 Wastren Advantage Inc., 120 Technology Drive, Idaho Falls, Idaho USA; 3 U. S. Bureau of Land Management, 400 West F Street, Shoshone, Idaho USA; 4 U. S. Bureau of Land Management, 1405 Hollipark Drive, Idaho Falls, Idaho USA; 5 Craters of the Moon National Monument and Preserve, P.O. Box 29, Arco, Idaho USA; 6 Department of Biology, Temple University, 1900 N. 12th St, Philadelphia, Pennsylvania USA

## Abstract

Bats of western North America face many threats, but little is known about current population changes in these mammals. We compiled 283 surveys from 49 hibernacula over 32 years to investigate population changes of Townsend’s big-eared bats (*Corynorhinus townsendii townsendii*) and western small-footed myotis (*Myotis ciliolabrum*) in Idaho, USA. This area comprises some of the best bat habitat in the western USA, but is threatened by land-use change. Bats in this area also face invasion by the pathogen causing white-nose syndrome. Little is known about long-term trends of abundance of these two species. In our study, estimated population changes for Townsend’s big-eared bats varied by management area, with relative abundance increasing by 186% and 326% in two management areas, but decreasing 55% in another. For western small-footed myotis, analysis of estimated population trend was complicated by an increase in detection of 141% over winter. After accounting for differences in detection, this species declined region-wide by 63% to winter of 1998–1999. The population fully recovered by 2013–2014, likely because 12 of 23 of its hibernacula were closed to public access from 1994 to 1998. Our data clarify long-term population patterns of two bat species of conservation concern, and provide important baseline understanding of western small-footed myotis prior to the arrival of white-nose syndrome in this area.

## Introduction

Bats of western North America face many threats. Disturbance during hibernation and destruction of hibernacula by humans have long been concerns for bat conservation in many areas^1–3^. Wind-energy development has led to extensive bat mortality^4–6^ and such development is expanding across the West. The recent arrival of white-nose syndrome, a deadly disease of hibernating bats^7,8^ in western North America^9^, is an important emerging threat to bats in this region. This disease has led to mass mortality in seven bat species in eastern and midwestern North America^10,11^, resulting in severe regional declines in several species^10,12^, and the potential for regional extirpation of some species within the next decade^13,14^. These threats make an understanding of population changes in western bats essential for the conservation of these mammals and their habitat^15,16^.

Estimation of long-term, regional population changes of bats is central for targeting management efforts^16–18^. Recent studies have evaluated long-term, regional population patterns of bats in eastern North America^10,12,19^, but such patterns remain poorly understood in western North America^20^. With the emergence of white-nose syndrome—as well as other threats—in the West, understanding population trends and establishing conservation baselines for bats in this region has become increasingly urgent^21^.

A western habitat important for bats is sagebrush (*Artemisia tridentata*) steppe^22–24^. Areas in southern Idaho are sagebrush-steppe habitat that include the largest, contiguous volcanic pseudokarst area in the USA^25^, which provides important cave habitat for bats. The intersection of important bat foraging and cave habitat accounts for high levels of bat use in this region, including one of the highest densities of hibernacula for Townsend’s big-eared bats (*Corynorhinus townsendii townsendii*) and western small-footed myotis (*Myotis ciliolabrum*) in North America^23,26^, as well as one of the highest densities of maternity roost sites for Townsend’s big-eared bats in the western USA^27^. Sagebrush steppe, however, has been altered by wildfires that have increased in intensity and extent^28^ as a result of climate change^29,30^ and exotic plant invasions^31^. Also, this habitat is under increasing threat from other land-use changes^32,33^. Alterations to sagebrush communities may affect not only foraging areas for bats, but also cave use by modifying microclimates of hibernacula within these communities^2,18,34^. Little is known, however, about the effects of such habitat alterations on bat populations in this area.

Our objective was to understand population changes of two western bat species. Specifically, we compiled a long-term, regional-scale dataset from bat monitoring, and used generalized additive mixed models (GAMMs)^35–37^ to understand population trajectories of Townsend’s big-eared bats and western small-footed myotis—both species of conservation concern^18,38^—while addressing factors that have previously complicated analyses of wildlife monitoring data^19,39^. We also evaluated the extent to which other factors, including cave characteristics and survey timing, might influence bat detection during surveys. Our analyses focused on count data from hibernacula surveys, which represent some of the longest, most reliable, and consistent datasets available for studies of population trajectories of bats^19^. Our results will clarify long-term trajectories of these populations, suggest possible causes for population changes, and provide baseline data in a region at risk for imminent invasion by white-nose syndrome.

## Methods

### Study areas

Our study areas comprised three management units (Big Desert, Sand Creek Desert, and Shoshone Desert) consisting of areas managed by two field offices of the US Bureau of Land Management (BLM) and by Craters of the Moon National Monument and Preserve. This land spans a 275 km straight-line distance in Bingham, Blaine, Bonneville, Butte, Clark, Fremont, Gooding, Lincoln, Minidoka, and Power counties in southern Idaho. Vegetation was sagebrush steppe. All hibernacula surveyed were simple lava-tube caves^22,23^, consisting mainly of one opening that were from 25 m to 2.1 km long, with ceiling heights up to about 10 m. Elevation at cave entrances ranged from 1,053 m to 1,875 m^22,40^. Caves were in cold deserts characterized by hot, dry summers and cold winters^22,41^. Most precipitation occurred during winter as snow and during spring as rain or snow^42^. Precipitation was at least twice as much in the Sand Creek Desert as in the Big and Shoshone deserts^43,44^.

The three management units differed in public access to caves. In the Big Desert, 25 caves were surveyed. Thirteen of those were closed to public access before our study and on land owned by Craters of the Moon National Monument and Preserve of the US National Park Service and the US Department of Energy, Idaho Operations Office^41^. Of the remaining 12 caves on land managed by the BLM, two caves had winter restrictions starting in 1998, and the others were open for public access. In the Sand Creek Desert, four caves were surveyed on land managed by the BLM. All of those caves were closed to public access during winter since 1997. Twenty caves were surveyed in the Shoshone Desert, 18 of which were on BLM land and two on private land. From 1992 to 2002, 12 of those caves that were used by both species of bats were gated or had winter restrictions limiting public access. The rest were open for public access. Overall, the Shoshone Desert had more recreationists visiting caves during winter than the other areas.

### Cave counts

Response data were bat counts collected during hibernacula surveys^45,46^ as part of long-term monitoring overseen by state or federal agencies between winter 1984–1985 and winter 2015-2016^22^. All surveys were conducted in one day, and mean survey date was 25 January (*SD* = 28 days). We only used counts that were conducted during hibernation (1 November to 31 March). All caves were surveyed in a consistent manner each year. Investigators visually identified and counted bats^46–48^, and used established protocols to minimize disturbing hibernating bats when conducting surveys^2,49^. Among surveys when observer data were recorded (*n* = 260), mean number of surveyors was 3 (*SD* = 1.2).

Townsend’s big-eared bats often occupy open areas of a cave during hibernation^22,50^ and are easily visible^51,52^. However, western small-footed myotis may wedge themselves in cracks when hibernating and are often not easily visible by researchers^45,53^. As a result, counts of that species were likely underestimated during surveys^54^. However, similar detection likely affected all surveys, and thus repeated surveys at a site were considered suitable for estimating population trends. Most bats we observed roosted singly; the remainder were in small clusters (median cluster size = 4 in both species, range for Townsend’s big-eared bats = 2 to 190 bats, range for western small-footed myotis = 2 to 33 bats), thus biases that occur in bat counts with large clusters (>1,000 individuals)^39^ were not a concern. While we surveyed all known hibernacula, additional hibernacula exist in our study areas. Thus, we focused on changes in relative rather than absolute bat abundance.

For each cave, we compiled data for variables that may predict bat counts. Those variables were management area, cave, survey year, day of winter of the survey, and cave length (m). Area (Big Desert, Sand Creek Desert, or Shoshone Desert) was included because of different land management and access to caves in those areas. Cave was a site identifier, because of repeated surveys at each cave. Inclusion of survey year enabled determination of across-year patterns (surveys in November 1984 to March 1985 were labeled as winter year 1985). Day of winter (with November 1 labeled as day 1) can influence occupation of caves by bats, as well as detection of bats by surveyors^17,52^. In 11 surveys only a range of dates was recorded (four surveys were conducted within a 46-day period in winter 1984-1985^22^, and seven surveys were conducted within eight-day periods in winter 1992–1993). We used the midpoint of the range as the survey date in those cases. In our study area, cave length may be an important variable for Townsend’s big-eared bats when these mammals select hibernacula^23^, because longer caves most likely provide more suitable microclimates for hibernation. We thus included that variable in the model selection for that species. We did not include cave length in model selection for western small-footed myotis, because cave length data were not available for several caves used by that species. We also examined the influence of number of observers and elevation at cave openings, but data exploration indicated that these were not important predictors of bat counts for either species. For this reason, because these data were missing for a number of surveys, and because missing data can bias model selection, we subsequently excluded those two variables.

The resulting dataset was 37,404 bats counted during 283 surveys in 49 caves from winter 1984–1985 to winter 2015–2016. Prior to analysis, we removed records for which species identification was uncertain (*n* = 5), and then separated the dataset by species. We then focused on repeatedly surveyed caves that served as hibernacula for ≥ 1 individual of the species. To avoid biases in model selection, we also removed any surveys with missing data for any predictor variable^55^. That process yielded a dataset for Townsend’s big-eared bats with 34,931 bats counted during 244 surveys in 39 caves, and a dataset for western small-footed myotis with 1,399 bats counted during 173 surveys in 23 caves. Mean number of surveys across our study per cave for Townsend’s big-eared bats was 6 (*SD* = 3.4) and for western small-footed myotis was 8 (*SD* = 3.4). The data that support the findings of this study are available from the Wildlife Diversity Program of the Idaho Department of Fish and Game, Boise, ID.

### Data analyses

To understand changes in bat populations over time, we used a multi-step process of model estimation and selection using GAMMs^35,36^ following protocols for bats developed by Ingersoll *et al*. (^12,19^). GAMMs enabled us to address several well-known challenges inherent in analyzing count data for bats, including non-normal error distributions, non-linear population patterns, short-term erratic changes in population pattern, non-independence of repeated samples at a cave, and inconsistencies in sampling timing and effort^12,19,56^. The global model for Townsend’s big-eared bats was equation (1).

1 $$\begin{array}{c}E[{y}_{t}]=g(u+{s}_{1}(year\,\times area)+{s}_{2}(day)+length+{v}_{jt})\\ \,\mathrm{Where}\,{y}_{it} \sim {\rm{Pois}}(E[{y}_{it}])\end{array}$$

*E*[*y*_*t*_] was the expected count at time *t*, *g* was the inverse of the selected link function (the natural logarithm, *ln*); *u* was the mean count; *year* was the winter year the cave was surveyed; *area* was the management area; *day* was the day of winter on which the cave was surveyed, *length* was the length of the cave, *v*_*jt*_ was a random effect of cave *j* at time *t*; and *s*_1_ and *s*_2_ were smoothing functions (cubic regression splines) for the interaction of *year* by *area* and the main effect of *day*. The global model for western small-footed myotis was the same except it excluded cave length. Both models assumed Poisson-distributed counts, as we did not find evidence of overdispersion.

We then reduced the global model using Akaike’s Information Criterion (AIC)^55,57^. Model reduction followed a stepwise process beginning with reduction of complex interaction terms, then smoothed terms, and finally simpler linear terms on the basis of AIC^19,58^. The random effect of *cave* was retained in all models to address non-independence of repeated surveys at the same cave.

We graphically rendered count trajectories and associated 95% confidence intervals (*CI*) from the best models^12,19,58^. When *day* was in selected models, we controlled for the effect of detection over winter months by fixing that variable at its median, then calculated trajectories as if they had all been sampled on the same day of winter^19^. When *area* was in selected models, trajectories were rendered by area, resulting in three trajectories for Townsend’s big-eared bats^12^. For this species, a separate overall regional pattern was also estimated from the same best model by averaging area-level trajectories. In line with inference appropriate to GAMMs^59^ and our emphasis on comparing changes in relative trend rather than estimating absolute abundances, we normalized each trajectory^12,19^ by dividing by the relative abundance at the beginning of the study period (i.e., the estimate for winter 1984–1985). This was done by management area when the *area* variable was retained in selected models. All analyses were completed in R version 3.1.3^60^ and the mgcv package version 1.8-4^61,62^. Alpha was set at 0.05 for all statistical tests.

## Results

Model selection for Townsend’s big-eared bats supported non-linear change in relative abundance over time that varied by management area; models without the smoothed *year* by *area* interaction term received little support (Table 1). The four best models (with a cumulative Akaike weight of 0.88) differed solely in the inclusion of linear *day* and *length*. There was slightly more support for the model including both of those terms, though evaluation of the best model indicated that both variables had only a marginally significant effect on relative abundance (*day*, *P* = 0.074; *length*, *P* = 0.088; Supplementary Fig. S1). All models with ΔAIC ≤2 produced count trajectories that were virtually identical (Supplementary Fig. S2).

**Table 1:** Information criteria for comparison of candidate models for Townsend’s big-eared bats from hibernacula surveys from winter 1984–1985 to winter 2015–2016 in southern Idaho, USA; K = number of parameters, AIC = Akaike’s information criterion, Δ_i_ = delta AIC, *w*_*i*_ = Akaike weight.

Model^a^	K	AIC	Δ_i_	*w*_*i*_
*s*(*year* x *area*) + *day* + *length*	11	633.7	0.0	0.27
*s*(*year* x *area*) + *day*	10	634.1	0.4	0.22
*s*(*year* x *area*) + *length*	10	634.1	0.4	0.22
*s*(*year* x *area*)	9	634.6	0.9	0.17
*s*(*year* x *area*) + *s*(*day*) + *length*	12	635.3	1.6	0.12
*year* x *area* + *day* + *length*	10	654.0	20.3	0.00
*s*(*year*) + *s*(*day*) + *area* + *length*	10	655.1	21.4	0.00
*year* x *area* + *s*(*day*) + *length*	11	656.6	22.9	0.00

^a^Fixed effects: *year*, the year the cave was surveyed; *area*, the management area in which the cave was located; *day*, the sequential day of winter during which the cave was surveyed; and *length*, cave length (m). Variables with *s*() notation are smoothed, otherwise all other variables are linear. Interaction terms indicated by x (e.g., *year* x *area*) and imply inclusion of main effects. A random effect of *cave* (a site identifier) is not shown, but was included in all models.

**Figure 1:**
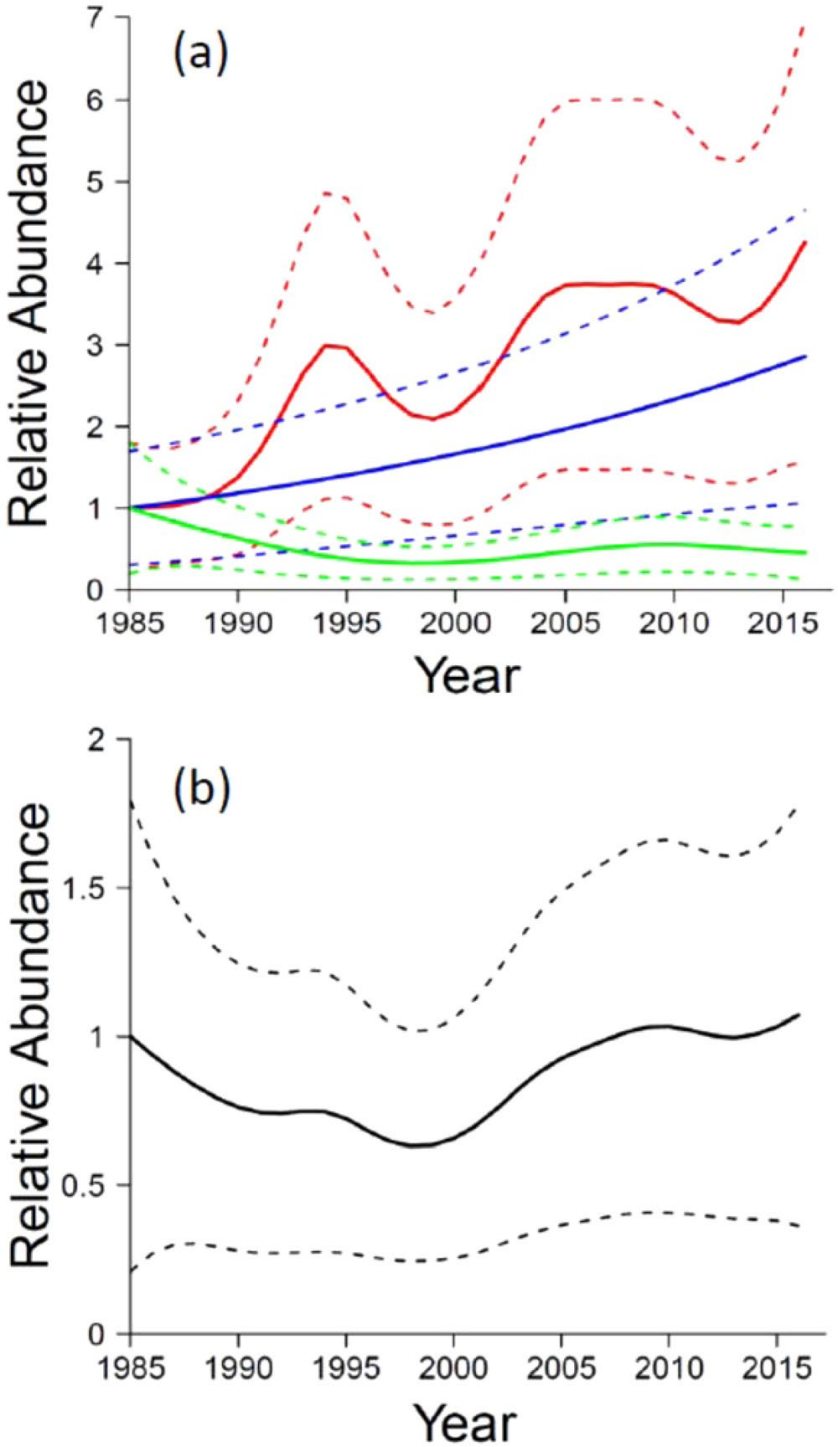
Relative abundances (solid lines) ± 95% *CI*s (dotted lines) of hibernating Townsend’s big-eared bats by year (**a**) across three study areas and (**b**) overall. Trajectories are from the best model, based on 244 surveys in 39 caves in southern Idaho, USA, from winter 1984–1985 (labeled as 1985 in the figure) to winter 2015–2016. In panel a, Big Desert = red line, Sand Creek Desert = blue line, and Shoshone Desert = green line.

Population patterns for Townsend’s big-eared bats differed considerably over time among management areas (*P* < 0.001 for all smoothed *year* by *area* interactions). By 2015–2016, abundance increased substantially in the Big Desert (326% higher than 1985 values) and in the Sand Creek Desert (186% higher), but decreased in the Shoshone Desert (55% lower than 1985 values) (Fig. 1a). Fluctuations were evident in trajectories in the Big and Shoshone deserts, but not in the Sand Creek Desert. Overall, across the study region, the population decreased 37% from 1985 to 1998, but had fully recovered by 2008 and was 7% higher at the end of the study period in 2016 than it was in 1985 (Fig. 1b).

**Figure 2:**
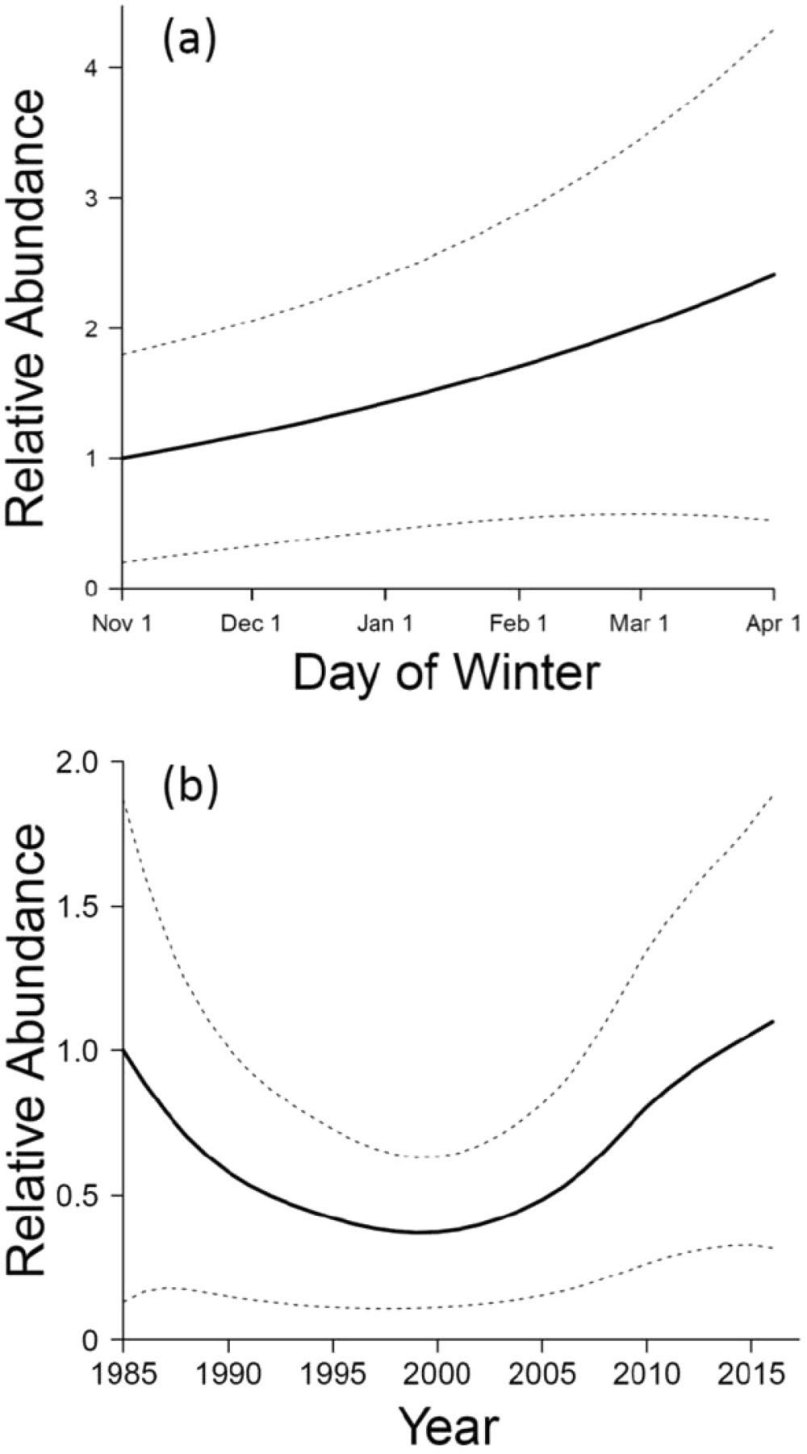
Relative abundances (solid lines) ± 95% *CI*s (dotted lines) of hibernating western small-footed myotis by (**a**) day of winter and (**b**) year. Trajectories are from the best model, based on 173 surveys in 23 caves in southern Idaho, USA, from winter 1984–1985 (labeled as 1985 in panel b) to winter 2015–2016.

Model selection for western small-footed myotis supported non-linear change in relative abundance over time that did not vary by *area*; models with a smoothed term for *year* and no interaction had a cumulative Akaike weight of 0.98 (Table 2). The best model had fairly strong support relative to other models, with the next-best models only differing in the inclusion or exclusion of linear terms. In the best model, counts of western small-footed myotis increased across winter months (*P* = 0.031), such that on average 141% more western small-footed myotis were counted during surveys at the end of winter (31 March) than at the beginning (1 November) (Fig. 2a).

**Table 2:** Information criteria for comparison of candidate models for western small-footed myotis from hibernacula surveys from winter 1984–1985 to winter 2015–2016 in southern Idaho, USA; K = number of parameters, AIC = Akaike’s information criterion, Δ_i_ = delta AIC, *w*_*i*_* = *Akaike weight.

Model^a^	K	AIC	Δ_i_	*w*_*i*_
*s*(*year*) + *day*	6	690.4	0.0	0.63
*s*(*year*) + *day* + *area*	8	693.4	3.0	0.14
*s*(*year*)	5	693.7	3.3	0.12
*s*(*year*) + *s*(*day*) + *area*	9	695.4	5.0	0.05
*s*(*year*) + *area*	7	695.8	5.4	0.04
*s*(*year* x *area*) + *s*(*day*)	11	698.0	7.6	0.01
*year* + *s*(*day*) + *area*	8	701.7	11.3	0.00
*year* + *day* + *area*	7	702.0	11.6	0.00

^a^Notation is as in footnote to Table 1. A random effect of *cave* (a site identifier) is not shown, but was included in all models.

Population pattern for western small-footed myotis differed considerably over time (smoothed *year*, *P* < 0.001). Relative abundance declined 63% from 1985 to 1999; however, abundance increased substantially thereafter, recovering fully by 2013–2014 (Fig. 2b). Greater variability in relative abundance of those bats was evident early and late in our study than in the middle.

## Discussion

Townsend’s big-eared bats have declined across parts of their range since the early 1900s, and are a species of conservation concern in the western USA^63,64^. Moreover, two eastern subspecies of these bats—the Virginia (*C*. *t*. *virginianus*) and the Ozark (*C*. *t*. *ingens*) big-eared bats, have been listed as endangered under the Endangered Species Act since 1979^63^. In our study areas, relative abundance of Townsend’s big-eared bats increased 326% in the Big Desert and 186% in the Sand Creek Desert since winter of 1984–1985, indicating the importance of these areas for the conservation of this species in western North America. Other studies in California and Washington using different analytical methods, covering smaller spatial scales, and shorter duration also documented increasing trends for this subspecies^17,48^. In the Shoshone Desert relative abundance of Townsend’s big-eared bats, however, decreased 55% since 1984–1985. One reason for that decrease may be the higher disturbance by recreational cavers during winter in the Shoshone Desert, where winter access is easier, and where many of the hibernacula are popular recreational caves, potentially resulting in numerous disturbances per winter. Overall, however, the trajectory for this species indicates a gradual decline occurred from the late 1980s to the late 1990s, followed by a more recent gradual recovery, consistent with the gating or winter recreation closure from 1994 to 1998 of 18 caves in our study areas.

Little is known about current population patterns of western small-footed myotis in western North America^65,66^. Our study is the first assessment of a long-term population trend for this species. Relative abundance decreased rapidly early in the study period, but the population has since recovered. The beginning of that increase was temporally concordant with the gating or winter recreation closure from 1994 to 1998 of 12 of the 23 caves used by western small-footed myotis in our study areas^67,68^.

Bat movement among hibernacula occurs both within and among years^24,52,69^, but the effects of bat movement on abundance estimates is reduced at large spatial scales, because low counts due to emigration from one site are counterbalanced by higher counts due to immigration to another^19^. Further, movement of Townsend’s big-eared bats appears quite limited among our study areas. In the Big Desert and Shoshone Desert, of 224 individuals that were observed in caves during winter, 95% returned to the cave in which they were banded, indicating a high degree of fidelity^71^. Bats in the Big Desert and Sand Creek Desert represent two distinct and divergent clades based on mitochondrial DNA from 114 individuals^70^. Additionally, banded Townsend’s big-eared bats in the Shoshone Desert (*n* = 385 individuals) and part of the Big Desert (*n* = 98 individuals) were never recaptured in hibernacula between those areas in a 2-year study^71^. Thus, our trajectories for Townsend’s big-eared bats should represent regional-scale population change rather than the effect of immigration and emigration.

In our study, confidence intervals around population estimates for each species were wide in some cases. That likely resulted from the low frequency of surveys (≥2 years apart) implemented to limit disturbance of hibernating bats, and from the year-to-year variation in counts typical of bat surveys^17,48,72^. For western small-footed myotis, fewer surveys early in the study period may also have contributed to wide confidence intervals. We interpret the widening of confidence intervals in that species late in the study period as resulting from spatial divergence in population pattern among sites, due to winter closures of hibernacula, potentially from the distribution of wildfires, or other factors not captured by our management area variable. Despite wide confidence intervals, changes over time in relative abundance were highly significant for both species.

Consistent survey timing is recommended to increase power to detect changes in relative abundance^19^, and where Townsend’s big-eared bats and western small-footed myotis co-occur, we recommend biologists standardize surveys during the end of hibernation (March in our study areas). Doing so will only minimally affect counts of Townsend’s big-eared bats, which did not vary significantly across the hibernation season; and will produce the highest count for western small-footed myotis, enabling easier detection of population changes. Additionally, researchers and surveyors should follow best practices to unobtrusively enter caves for legitimate scientific purposes only once in a survey year to limit the amount of disturbance to hibernating bats. Late winter also corresponds with the best time for surveillance of western small-footed myotis for white-nose syndrome, due to the progressive nature of infection over the winter^73–75^.

Our results clarify long-term population patterns of two bats of conservation concern in sagebrush-steppe communities^32,33^, suggest potential causes of change for these species, and can guide future surveys. More than 1,500 lava-tube caves exist in southern Idaho^23^; undoubtedly, more caves are used as hibernacula in our study areas than we identified^22,23,71^. Researchers in our study, however, were able to consistently access surveyed caves in winter compared with other caves in our study areas, which led to our long-term data set. The bats we studied also have estimated generation times of 5.8 years (western small-footed myotis) and 3.5 years (Townsend’s big-eared bats)^76^; therefore, our results provide an essential, long-term baseline of relative abundance to quantify future potential declines in these species, including from disturbance, wildfires, and land-use changes near these caves. Additionally, our results are particularly relevant to the recent arrival of white-nose syndrome in the western USA^9^, within 750 km from our study areas. General conditions of humidity and temperature exist for growth of *Pseudogymnoascus destructans* (the fungus that causes white-nose syndrome) in caves in southern Idaho and across many areas of the West^20,23^. High infection rates of wintering populations of *Myotis* spp. in areas currently affected by white-nose syndrome^14,16,77^ heighten concern, especially for western small-footed myotis. For all of these reasons, continued tracking of populations of western bats is critical^21^.

## Electronic supplementary material

Supplementary information
