# Supplementary material for: Long-term bat abundance in sagebrush steppe
Source: Sci Rep. 2018 Aug 16;8:12288. doi: 10.1038/s41598-018-30402-z (PMC6095839; doi:10.1038/s41598-018-30402-z)
Supplement: Supplementary file 1 — Supplementary information [file 41598_2018_30402_MOESM1_ESM.docx]

**Long-term bat abundance in sagebrush steppe**

Jericho. C. Whiting^*^, Bill. Doering, Gary. Wright, Devin. K. Englestead, Justin. A. Frye, Todd. Stefanic, & Brent. J. Sewall

**Supplementary information.** Further exploration of models for Townsend’s big-eared bats.

In the best model of population trajectory of Townsend’s big-eared bats (Table 1), counts decreased linearly across winter months, such that on average 23.5% fewer Townsend’s big-eared bats were counted during a survey at the end of winter (31 March) than at the beginning (1 November) (Fig. S1a). Variation was high, however (Fig. S1a), and the linear variable for *day* was only marginally significant (*P* = 0.074). Counts of this species also increased with cave length, such that on average, 460% more Townsend’s big-eared bats were counted in the longest cave in the region (2,140 m) than in the shortest (25 m) (Fig. S1b). Variability was considerable for longer caves, however (Fig. S1b), and overall the linear variable for *length* was also only marginally significant (*P* = 0.088).

In addition to the best model of population trajectory for Townsend’s big-eared bats, four additional models received some support (ΔAIC ≤ 2; Table 1). Those models did not differ in interactions (Table 1). Graphical representation (Fig. S2) revealed that trajectories of all four alternate models were nearly identical to trajectories from the best model (Fig. 1).

**Supplementary Information Figure legends**

**Fig. S1**. Relative abundances (solid lines) ± 95% *CIs* (dotted lines) of hibernating Townsend’s big-eared bats by (a) day of winter and (b) cave length. Figures are extracted from the best model for Townsends’s big-eared bats, and show variables that were marginally significant in the model (0.05 ≤ *P* ≤ 0.10). Data are from 244 surveys in 39 caves in southern Idaho, USA.

**Fig. S2**. Relative abundances (solid lines) ± 95% *CI*s (dotted lines) of hibernating Townsend’s big-eared bats across 3 study areas by year, according to four alternate models with some support (ΔAIC ≤ 2) relative to the best model shown in Fig. 1 in the main text. Represented are models for (a) *s*(*year* x *area*) + *day*, (b) *s*(*year* x *area*) + *length*, (c) *s*(*year* x *area*), and (d) *s*(*year* x *area*) + *s*(*day*) + *length*. Model notation follows Table 1, and each model also includes a random effect of *cave* (a site identifier). Trajectories are from 244 surveys in 39 caves in southern Idaho, USA, from winter 1984-1985 (labeled as 1985 in the figure) to winter 2015-2016. Big Desert = red line, Sand Creek Desert = blue line, and Shoshone Desert = green line.

**Fig. S1**.

**Fig. S2**.
